# Deployment of workforce in global health: what should be the priorities for China?

**DOI:** 10.1186/s41256-021-00208-0

**Published:** 2021-07-06

**Authors:** Xuejiao Ma, Wei Ding, Yingjun Qian, Shenning Lu, Bei Wang, Qiuli Xu, Duoquan Wang, Yayi Guan, Ning Xiao, Xiaonong Zhou

**Affiliations:** 1 National Institute of Parasitic Diseases, Chinese Center for Disease Control and Prevention (Chinese Center for Tropical Diseases Research), No. 207 Ruijin Er Road, Huangpu District, Shanghai, 200025 China; 2 NHC Key Laboratory of Parasite and Vector Biology, Shanghai, 200025 China; 3 WHO Collaborating Centre for Tropical Diseases, Shanghai, 200025 China; 4 National Center for International Research on Tropical Diseases, Shanghai, 200025 China; 5 School of Global Health, Chinese Center for Tropical Diseases Research, Shanghai Jiao Tong University School of Medicine, Shanghai, 200025 China

**Keywords:** Global health, Chinese workforce, Priorities, Competencies, Deployment

## Abstract

**Background:**

China has increasingly emerged as an important player in global health. However, compared to developed countries, China still lacks a sufficient health workforce for global health engagement with the necessary competencies required. The world has recognized that to solve global health issues, the role of China needs to be strengthened. The priorities for the deployment of the Chinese workforce in global health remain unclear. This study aims to identify the priorities of the deployment of Chinese global health workforce by exploring the core competencies for Chinese global health workforce, factors influencing the deployment and the approach of deployment.

**Methods:**

Quantitative descriptive statistical analysis was applied to analyze the quantitative data. A total of 148 key respondents from 10 provinces in China conducting global health projects over the last 3 years were selected as the study subjects. A structured questionnaire was developed to collect the data on four aspects, including general information, core competencies, factors influencing deployment, and mode of deployment. The questionnaire was distributed to the respondents through an online survey. All original data were exported to Microsoft Excel 2010 to calculate the frequencies and percentages of each option. A descriptive analysis was carried out of the priorities of deployment of the Chinese global health workforce.

**Results:**

More than half of the respondents (51.4%, 76/148) regarded “communication” as the most important competency of the Chinese global health workforce, while a large proportion of participants from Chinese embassies (50.0%, 6/12) and international organizations (75.0%, 12/16) believed that “professional skills” were paramount. In addition, 58.1% (86/148) of the participants agreed that incentive factors (salary, professional position, etc.) were the main factors that influenced deployment, whereas 75% (12/16) of participants from international organizations emphasized “security” as the most important determinant. In addition, 60.8% (90/148) of the participants thought that the deployment of staff should be based on the needs of the global health project implementation.

**Conclusions:**

This study highlights the deployment priorities of the Chinese global health workforce, including strengthening communication and professional skills, focusing on personal security and incentives, and catering to the project implementation. This study also highlights the importance of Chinese agencies in developing global health mindsets through global health practices and proactive integration within the global community.

**Supplementary Information:**

The online version contains supplementary material available at 10.1186/s41256-021-00208-0.

## Background

China has always been a strong advocate and practitioner of global health, and has demonstrated a firm commitment to its improvement [1]. One of the aims of China’s “Belt and Road Initiative” was to improve the health of the populations of the countries alongside [2]. As part of its commitment to the South-South Cooperation, Chinese President Xi Jinping proposed the China-Africa Public Health Cooperation during his visit to Africa in December 2015 [3]. Since 1963, China has built more than 100 health facilities with an estimated 23,000 global health workers dispatched overseas [2]. Recently, China’s engagement in global health is receiving significant attention and recognition in the global health arena [4]. China has committed to providing assistance through existing channels such as the Forum on China-Africa Cooperation (FOCAC), and the China International Development Cooperation Agency (CIDCA) was established to support a greater Chinese role in global health [4]. China plans to extend its global health initiatives such as the Health Care Initiative launched at the 2018 FOCAC Beijing Summit. China has also been working with the African Union and other international partners to support the establishment of the Africa Centres for Disease Control and Prevention (CDC) [5]. All these highlight the importance and necessity of health workforce for global health initiatives of China.

However, compared to some developed countries, China is behind in the recruitment and deployment of its global health workforce [6]. This may be attributed to shortage of competent workforce for global health in China and lack of stronger cooperation with international agencies [6, 7]. China is already facing shortage of public health personnel with 0.63 public health personnel per 1000 population since 2018 and worst still, there is a significant shortage of experienced senior health professionals in the country [6]. Consequently, allocating and deploying Chinese health professionals for global health initiatives seems a difficult option under the present circumstances.

The issue for deployment is not just the scarcity of health professionals but the necessary “qualified capacity” needed for global health initiatives [8]. There are limited number of training schools in China that offer professional global health courses and furthermore, the quality of training is not at par with the expectations of the international communities [9, 10]. The above instances show, in order to enhance its global health initiatives, China has to address the above informed challenges for its global health initiatives.

In scenarios of scarcity of skilled and lesser number of health professionals for global health initiatives, China needs to consider the effective deployment of its global health workforce. Global health workforce are health professionals working globally, who are committed to placing a priority on improving health and achieving equity in health for all people worldwide [11]. While China aims to deploy such workforce, yet the priorities for the deployment of Chinese global health workforce for global health initiatives remain unclear. This study aims to identify the priorities for the deployment of Chinese global health workforce by analyzing the (i) preferred core competencies for deployment; (ii) factors influencing deployment and (iii) preferred mode of deployment.

## Methods

### Study design & sample

Quantitative descriptive statistical analysis was applied to obtain the most important sensory terms and analyze the quantitative data [12]. Purposive sampling was used in the study. The study subjects were selected by the researcher in order to answer the specific research questions [13]. The respondents consisted of a total of 148 key stakeholders from 46 organizations directly involved in global health. The 46 organizations included three international organizations and 43 Chinese organizations. The 43 Chinese organizations were from Chinese domestic administrative agencies, Chinese professional institutes, Chinese embassies, and foreign aid medical teams from 10 Chinese provinces. The selection criteria were as follows: (i) in Chinese domestic administrative agencies, 3–4 staff members engaged in international cooperation were selected (given the limited number); (ii) in Chinese professional institutes, 1–2 relevant leaders and 2–3 key members were selected from each institute involved in the global health program; (iii) in the Chinese embassies in Africa where the corresponding medical teams were located, 1–2 relevant counselors from each embassy were selected; (iv) in Chinese medical teams in Africa, 1–2 team leaders and 2–3 core members were selected from each team; (v) in international organizations, 3–4 project leaders and participants were selected from each institute. The selection criteria for each respondent were: (i) responsible for or participating in the management, implementation, monitoring, and evaluation of global health projects; (ii) responsible for or participating in global health projects for more than 6 months; (iii) a mid-level professional title or higher. Those who met all three criteria were the targeted respondents. The respondents’ demographic information is presented in Table 1.

**Table 1:** Demographic information on respondents from five organizations related to China’s global health engagement

Variable	Description	Frequency (N)	Percentage (100%)
Gender	Male	96	64.9
	Female	52	35.1
	Total	148	100
Organizations	Domestic administrative agencies	38	25.7
	Professional institutes	54	36.5
	Embassies	12	8.1
	Foreign aid medical teams	28	18.9
	International organizations	16	10.8
	Total	148	100
Related experience (years)	< 3	14	9.5
	3 ~ 5	80	54.1
	5 ~ 10	30	20.3
	> 10	24	16.1
	Total	148	100

### Questionnaire development

The questionnaire was developed by 10 senior professionals with at least 5 years of experience in global health. Three rounds of expert consultations were carried out before the pre-tests. The questionnaire was then piloted among 15 respondents with at least 3 years’ experience in global health. The questionnaire was further revised based on the respondents’ feedback. The final questionnaire consisted of four parts: general information; core competencies; factors influencing deployment; preferences for deployment.

### Data collection

The survey was approved by the National Institute of Parasitic Diseases, Chinese Center for Disease Control and Prevention Ethical Review Committee (Approval No. 2021010). It was conducted from 27 August to 31 December, 2020. The investigators communicated with targeted respondents via WeChat and sent them the specific link to the e-questionnaire (https://www.wjx.cn/). Participation in the survey was fully voluntary, and written informed consent was obtained from each participant. The language for data collection was Chinese. The objectives of the study, the confidentiality of individual information, and other ethical considerations mentioned in the survey guidelines were explained to the participants prior to data collection [14]. In total, 148 questionnaires were collected.

### Data processing and analysis

A descriptive analysis was used for the deployment priorities of the Chinese global health workforce. The data were represented by frequency, percentage and composition ratio for statistical description [15]. All original data were exported to Microsoft Excel 2010 to automatically calculate the frequency and related percentage of every answer to each question. The percentage was calculated based on the following formula: percentage = number of people choosing a particular option/number of people who completed the question * 100%. The higher the percentage, the higher the number of people who chose the option.

## Results

### Demographic characteristics of participants

Out of the 150 questionnaires distributed, 148 were collected with an effective response rate of 98.7%. Table 1 shows the demographic characteristics of the study respondents by number and percentage. Among the participants, 62.2% (92/148) were from Chinese organizations in charge of deploying the global health workforce, 27% (40/148) were from Chinese embassies and Chinese foreign aid medical teams, and 10.8% (16/148) were from international organizations with extensive experience in global health. Majority of the respondents were males (64.9%). Regarding the global health experience, 90.5% (134/148) of participants had more than 3 years’ experience in global health and 16.1% (24/148) had over 10 years’ experience. More than half of the respondents had a 3–5 years’ experience of working in global health initiatives.

### Preferred core competencies for deployment

**Fig. 1:**
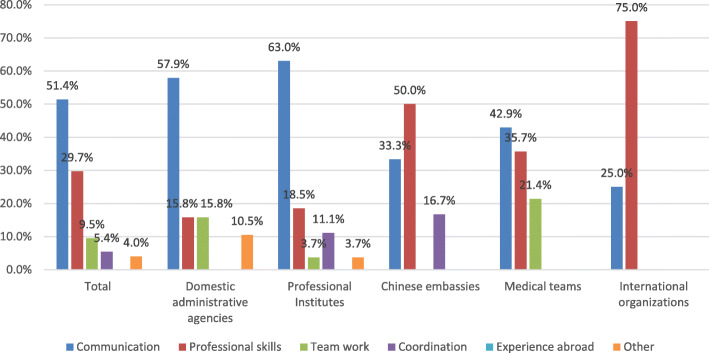
Core competencies required for Chinese global health workforce according to five different organizations

Figure 1 shows the percentage of core competencies stated by five different organizations in answering to the question, “What is the most important competency for the Chinese global health workforce”. More than half of the respondents (51.4%, 76/148) believed that “communication” was the paramount competency for the global health workforce followed by “professional skills” (29.7%, 44/148). On the other hand, respondents from Chinese embassies and international organizations regarded “professional skills” as their preferred core competency, accounting for 50.0% (6/12) and 75.0% (12/16) respectively. The survey results indicate that the global health workforce are expected to have comprehensive competencies. Among these competencies “communication” and “professional skills” predominated in the responses followed by “team work”, “coordination” and “experience abroad”.

### Factors influencing deployment

**Fig. 2:**
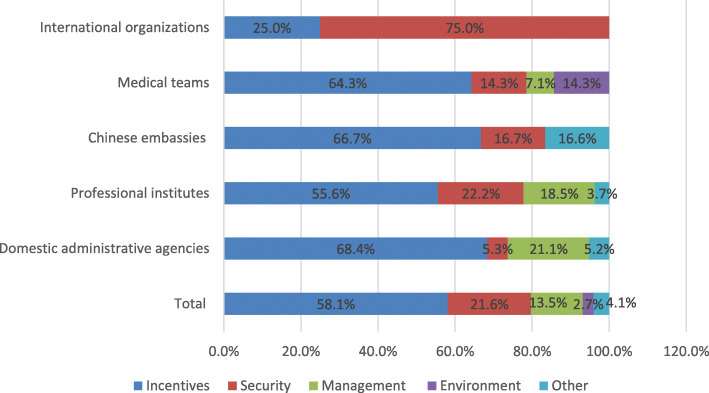
Factors influencing the deployment of the Chinese global health workforce according to five different organizations

Figure 2 represents the factors influencing deployment of global health workforce by five organizations in answering the question of “What is the foremost factor influencing deployment of Chinese global health workforce?” “Incentives” for global health workforce were regarded as the most important motivational factor in influencing deployment for global health initiatives by organizations except international organizations. However, a majority of respondents 75.0% (12/16) from international organizations preferred “security”. “Security” refers to the state of physical safety in the workplace including personal and property safety and all precautions taken to guard against dangers, risks, etc. to protect global health workforce [16]. Therefore, “incentives” and “security” for global health workforce might be emphasized by the policy makers of China when deploying for global health initiatives.

### Deployment preferences

**Fig. 3:**
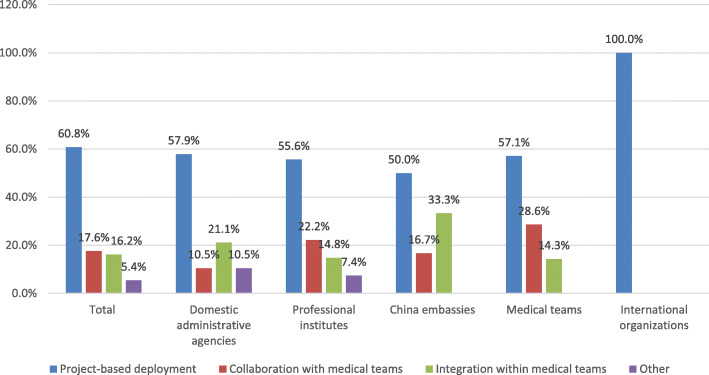
Deployment modes* of Chinese global health workforce according to five different organizations. *Three alternative modes of deployment of Chinese global health workforce. Project-based deployment refers to independently aligning with the global health workforce with project needs. Collaboration with medical teams refers to working closely with medical teams. Integration within medical teams refers to deploying the global health workforce as members of medical teams

Figure 3 shows the responses of the respondents regarding the mode of deployment of the Chinese global health workforce based on the question of “Which preferable ways do you think Chinese health workforce should be deployed for Chinese health initiatives globally?” The responses shows that 60.8% (90/148) of the respondents believed that project-based deployment was the best. The present findings suggest that most of the respondents prefer the deployment of Chinese health workforce to be customized based on project needs.

## Discussion

This is the first survey-based study on the deployment priorities of the Chinese global health workforce. The most important findings are the three priorities identified in the study: (i) the need for communication and professional skills; (ii) the role of incentives and security for the deployment of Chinese global health workforce; (iii) project-based deployment as the preferred approach. Another important finding was the overwhelming proportion of respondents from international organizations with different opinions regarding the three aspects of deployment.

### Strengthening communication and professional skills

As shown in Fig. 1, professional skills were regarded as the top priority by respondents from international organizations (75.0%) and Chinese embassies (50.0%). However, the majority of respondents from administrative agencies who deploy global health professionals, considered “communication” as the most important competency. Communication between the Chinese workforce and their local working partners remains a barrier that needs to be addressed in order to improve China’s global health engagement [16]. This is in line with previous studies that recognized language and communication as one of the main challenges for the Chinese global health workforce and that “both sides (Chinese and African professionals) need to strengthen communication to overcome language and cultural barriers” [17, 18].

A high proportion of respondents from international organizations and Chinese embassies regarded “professional skills” as a priority, while Chinese professional institutes attached less importance to it. Different stakeholders have different perceptions on this topic. International organizations and Chinese embassies emphasized China’s contribution to global health [8], while Chinese administrative agencies and professional institutes took “professional skills” for granted, believing that communication skills and limited field practices are the most challenging aspects of global health engagement [19]. This suggests that Chinese global health agencies should develop global health mindsets through global health practices and proactive integration within the global community.

### Stressing personal security and incentives

A very high proportion (75%) of participants from international organizations regarded “security” as the most important factor in deployment, while most Chinese organizations attached less importance to it (Fig. 2). This sharp contrast is because international organizations have different work environments and understanding of local contexts compared to Chinese global health agencies, i.e. international stakeholders have more experience of working abroad, while Chinese agencies lack local field practices and might ignore the importance of security for the Chinese workforce. Previous research also shows that security and traffic accidents were the major challenges for China’s overseas medical teams [20]. International organizations have high regard for security in global health, e.g. World Health Organization highlights the importance of job security and occupational safety in the *Global strategy on human resources for health: Workforce 2030* [21]. This suggests that Chinese administrative agencies should pay more attention to the security aspects of Chinese global health professionals when deployed abroad.

More than half of the respondents (58.1%) from the five organizations found incentives to be the principal factor, influencing the willingness of individuals to engage in global health. This might be because there are limited career paths or lack of incentives for global health work in China. In addition, the current human resource policies, such as payments, titles and professional promotions need to be reconsidered for motivating and retaining the Chinese global health workforce [16, 22]. It is thus recommended to take into consideration of staff’s personal security, and also to offer opportunities to achieve personal development while performing overseas global health assignments [20].

### Catering to the project implementation

Regarding the mode of deployment preferences, almost two thirds of respondents (60.8%), especially those from international organizations considered that the Chinese global health workforce should be project-based. Given that most of China’s global health professionals are deployed temporarily for major global health events without any standardization [23], discussion has been held regarding the deployment of the global health workforce together with China’s foreign aid medical teams. The latter has accumulated over 50 years’ experience providing medical service abroad. However, our study found that most respondents, including all the respondents from international organizations with more experience in global health, believed that the deployment should depend on the tasks required or the specific project. There are two possible explanations for this: firstly, the work of public health professionals, for example, mainly regards disease prevention and behavior change in communities and individuals. In contrast foreign aid medical teams work in fixed locations such as hospitals [24]. Secondly, targeted deployment in line with project needs is more effective at reaching project goals. A Chinese global health workforce cannot therefore be simply integrated within foreign aid medical teams but needs to adapt to the specific needs of different health projects.

### Limitation of this study

This research has two main limitations. Due to travelling restrictions caused by the COVID-19 Pandemic, only 8.1% of participants were from Chinese embassies and 10.8% were from international organizations. The limited participation of embassy and international staff may have led to a skewed representation of the study’s findings. This study was also limited by the small sample size, which might contribute potential bias to the results. These limitations could be addressed by including more respondents with China’s growing participation in global health.

## Conclusions

In this study, we have identified three priorities in the deployment of the Chinese global health workforce. Priority one is to fill the competency gaps, especially in terms of communication and professional skills, and also cultivate an interdisciplinary workforce, in order to meet the international standards for global health activities [25]. Priority two is to emphasize personal security and incentives in order to motivate more skilled people to engage in global health. Priority three is to consider project-based deployment in line with project needs. This study also highlights the needs for Chinese agencies to foster global health mindsets by accumulating global health experience and proactive integration within the global community. Further studies are necessary in order to contribute more in depth to China’s global health pathway.

## Supplementary Information

**Additional file 1.**

## Data Availability

Please contact the corresponding author for data requests.

## Abbreviations

COVID-19: Coronavirus disease 2019
FOCAC: Forum on China-Africa Cooperation
CIDCA: China International Development Cooperation Agency
CDC: Centres for Disease Control and Prevention
