# Supplementary material for: Deployment of workforce in global health: what should be the priorities for China?
Source: Glob Health Res Policy. 2021 Jul 6;6:22. doi: 10.1186/s41256-021-00208-0 (PMC8258270; doi:10.1186/s41256-021-00208-0)
Supplement: Supplementary file 1 — Additional file 1. [file 41256_2021_208_MOESM1_ESM.docx]

**Appendix 1**

**Questionnaire on the skills needed for deployment of professionals for global health assistance of China (Translated from Chinese)**

1. **Basic information**

1. Name 2. Gender (Male, Female)

3. Organization 4. Professional titles

5. Years of work in global health

1. **Questionnaire content**

6. What is the most important competency^[[1]](#footnote-1)^ for the Chinese global health workforce in the short term?

A. Communication^[[2]](#footnote-2)^ B. Coordination^[[3]](#footnote-3)^ C. Experience abroad^[[4]](#footnote-4)^

D. Professional skills^[[5]](#footnote-5)^ E. Teamwork^[[6]](#footnote-6)^ F. Other

7. What is the most important competency for Chinese global health workforce in the long term?

A. Communication B. Coordination C. Experience abroad

D. Professional skills E. Teamwork F. Other

8. What is the most important factor influencing^[[7]](#footnote-7)^ the deployment of Chinese global health workforce in the short term?

A. Incentives (salary, professional title, etc.)^[[8]](#footnote-8)^ B. Work environment^[[9]](#footnote-9)^

C. Security^[[10]](#footnote-10)^ D. Management way^[[11]](#footnote-11)^ E. Other_________________________

9. What is the most important factor influencing deployment of Chinese global health workforce in the long term?

A. Incentives (salary, professional title, etc.) B. Work environment

C. Security D. Management way E. Other_________________________

10. Which preferable ways^[[12]](#footnote-12)^ do you think Chinese health workforce should be deployed for Chinese health initiatives globally in the short term?

A. Project-based deployment^[[13]](#footnote-13)^

B. Collaboration with medical teams^[[14]](#footnote-14)^

C. Integration within medical teams^[[15]](#footnote-15)^

D. Other__________________________________

11. Which preferable ways do you think Chinese health workforce should be deployed for Chinese health initiatives globally in the long term?

A. As members of China’s foreign aid medical team

B. According to the needs of the implementation of global health projects

C. Collaboration with foreign aid medical team

D. Other__________________________________

This is the end of the questionnaire. Thank you for your support!

1. Based on the pretest, core competencies of China’s global health workforce refer to the components of communication, coordination, experience abroad, professional skills, team cooperation and others. [↑](#footnote-ref-1)
2. Communication refers to exchanging information freely and expressing ideas effectively with working partners overseas and a reasonable level of English competency is often important in this condition. [↑](#footnote-ref-2)
3. Coordination refers to making different groups of people to work together in an efficient and organized way. [↑](#footnote-ref-3)
4. Experience abroad means that one has worked or studied over a period of time overseas. [↑](#footnote-ref-4)
5. Professional skills refer to having specialized knowledge and practices in one’s own field. [↑](#footnote-ref-5)
6. Teamwork refers to working with other people to achieve a shared goal. [↑](#footnote-ref-6)
7. Based on the pretest, factors influencing the deployment include incentive policies, work environment, security, management way and others. [↑](#footnote-ref-7)
8. Based on the pretest, incentives refer to things that encourage China’s global health workforce to work overseas, including salary, professional title, home leave, vacation, medical insurance, housing, etc. [↑](#footnote-ref-8)
9. Work environment refers to the surroundings in workplace overseas. [↑](#footnote-ref-9)
10. Security refers to the state of physical safety in the workplace including personal and property safety and all precautions taken to guard against dangers, risks, etc. to protect global health workforce. [↑](#footnote-ref-10)
11. Management way refers to the way of leadership to administer and regulate China’s global health workforce overseas. [↑](#footnote-ref-11)
12. Mode of deployment refers to three alternative ways of deployment with no overlap, namely, project-based deployment, collaboration with medical teams and integration within medical teams. [↑](#footnote-ref-12)
13. Project-based deployment refers to deploying global health workforce independently aligning with the project needs. [↑](#footnote-ref-13)
14. Collaboration with medical teams refers to deploying workforce in cooperation with medical teams. [↑](#footnote-ref-14)
15. Integration within medical teams refers to deploying them as a member of medical teams. [↑](#footnote-ref-15)
